# Medication Adherence Interventions for Older Adults: A National Mixed‐Methods Study of Implementation Across Italian Healthcare Settings

**DOI:** 10.1111/jgs.70596

**Published:** 2026-08-03

**Authors:** Sara Mucherino, Luca Pasina, Manuela Casula, Monia Donati, Carlotta Lunghi, Elena Olmastroni, Valentina Orlando, Emanuel Raschi, Stefano Scotti, Marco Salluzzo, Elisabetta Poluzzi, Alessandro Nobili, Enrica Menditto

**Affiliations:** ^1^ Department of Pharmacy University of Naples Federico II Naples Italy; ^2^ Center of Pharmacoeconomics and Drug Utilization Research (CIRFF) University of Naples Federico II Naples Italy; ^3^ Health Policies Research Department, Laboratory of Clinical Pharmacology and Appropriateness of Drug Prescription Istituto di Ricerche Farmacologiche Mario Negri IRCCS Milan Italy; ^4^ Epidemiology and Preventive Pharmacology Service (SEFAP), Department of Pharmacological and Biomolecular Sciences University of Milan Milan Italy; ^5^ IRCCS MultiMedica Sesto San Giovanni Italy; ^6^ Department of Medical and Surgical Sciences University of Bologna Bologna Italy; ^7^ Population Health and Optimal Health Practices Research Unit, Centre de Recherche du CHU de Québec‐Université Laval Québec City Canada; ^8^ Department of Life Sciences, Health and Health Professions Link Campus University Rome Italy

**Keywords:** adherence interventions, medication adherence, mixed‐method study, older adults, stakeholder engagement

## Abstract

**Background:**

Medication non‐adherence is common among older adults and represents a major challenge for healthcare systems. Effective interventions are needed to support adherence and inform quality‐improvement efforts.

**Objective:**

To assess how medication‐adherence interventions for older adults are currently implemented in Italy and to identify care‐delivery priorities improving quality and feasibility across healthcare settings.

**Methods:**

We conducted a mixed‐methods study within the Eldercare project. We administered a national benchmarking survey (May–August 2024) to healthcare professionals and patient representatives from scientific societies, professional orders, public institutions, universities, and patient associations. We ran a nested Strengths, Weaknesses, Opportunities and Threats (SWOT) survey (January–March 2025) with key informants. We summarized quantitative responses, compared distributions across professional roles (*χ* ^2^/Fisher's exact tests), and analyzed SWOT free‐text using a hybrid deductive–inductive Framework Method.

**Results:**

In the benchmarking survey (*n* = 340), Respondents reported that adherence is mainly addressed during routine consultations (78.5%) and most often relied on direct patient‐provider communication (51.5%). Respondents reported that monitoring is primarily based on clinical data (55.3%) and access to dispensing/prescription data and digital tools was less frequent (generally ≤ 50%). Respondents rated patient‐centered strategies as highly important (e.g., caregiver involvement 94.4%, shared goal setting 90.3%, individual counseling 87.6%), but feasibility ratings were consistently lower. The most frequently reported unmet needs were limited patient awareness (68.8%) and poor coordination among professionals (60.3%). Key barriers included high numbers of daily medicines (69.1%) and lack of clear information on prescribed therapies (64.7%). In the SWOT survey (*n* = 14), strengths included continuity and communication, while weaknesses and threats focused on fragmented interprofessional collaboration, limited training, low digitalisation, and scarce financial/institutional support.

**Conclusions:**

Medication‐adherence support for older adults in Italy remains largely consultation‐based and variably resourced. Strengthening interprofessional coordination, training, and use of digital/administrative data may help develop more structured and scalable adherence‐support pathways as a quality‐improvement model of care.

## Summary

- Key points
  ○ Adherence support for older adults in Italy is still largely built on direct clinician–patient communication during routine care, with limited use of shared data and digital tools to enable systematic monitoring and coordinated follow‐up across settings.
  ○ Experts highlight continuity of care, strong patient‐provider communication, and nurse‐led coordination as key strengths, and see patient engagement, targeted training, and policy‐driven investment in digital solutions as major opportunities; fragmentation of interprofessional collaboration, limited training, and low digitalisation remain the main weaknesses and threats.
  ○ Compared with countries with funded, structured adherence services, Italy can strengthen implementation of adherence interventions by investing in recognized interprofessional pathways and interoperable data to support systematic monitoring and follow‐up
- Why does this paper matter?
  ○ This national mixed‐methods study provides the first system‐level assessment of medication‐adherence interventions for older adults in Italy. By combining benchmarking and stakeholder Strengths, Weaknesses, Opportunities and Threats (SWOT) analysis, it identifies actionable priorities to make adherence support more feasible and scalable across healthcare settings.

## Graphical abstract

Implementing medication adherence interventions for older adults across healthcare‐system levels.

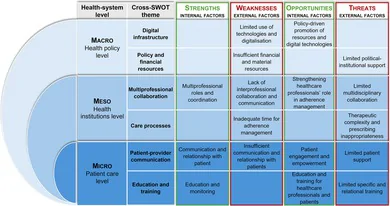

## 1 Introduction

Population aging is one of the most significant challenges facing healthcare systems worldwide [1]. Older adults frequently live with multiple chronic conditions requiring complex pharmacological regimens, resulting in widespread polypharmacy [2]. In this context, medication adherence is a critical determinant of treatment effectiveness, patient safety, and healthcare sustainability [3]. Cognitive and functional decline, combined with polypharmacy, compromise patients' ability to follow prescribed treatment regimens. Improving medication adherence is therefore a priority for aging health systems.

However, supporting medication adherence in older adults remains challenging in routine clinical practice. Time constraints during clinical encounters, limited interprofessional collaboration, and the underuse of health information systems may hinder the systematic implementation of adherence‐support strategies. Evidence suggests that multicomponent, patient‐centered, and interprofessional interventions are more effective than isolated actions [4]. However, despite growing international attention to medication adherence, the systematic integration of adherence assessment and support strategies into everyday clinical practice remains limited, particularly for older adults receiving multiple therapies [5].

Even when adherence‐enhancing interventions are identified [6], their organizational feasibility varies substantially across healthcare systems and care settings [7]. Evidence on how these interventions are implemented in real‐world healthcare contexts remains limited. In Italy, little is known about how medication adherence is currently managed, monitored, and supported across regions and professional groups, or which strategies are perceived as most relevant and feasible by healthcare professionals and patients. Furthermore, organizational factors, including strengths, weaknesses, and contextual barriers, that may influence the implementation and scaling‐up of adherence‐enhancing interventions remain poorly characterized. To address these gaps, the present study aimed to assess how medication‐adherence interventions for older adults are currently implemented in Italy and to identify priorities to improve their quality and feasibility across healthcare settings. Accordingly, we framed this work as a models‐of‐care quality‐improvement assessment focused on implementation, feasibility, and system‐level levers rather than clinical effectiveness.

## 2 Materials and Methods

### 2.1 Study Design

This study adopted a mixed‐methods design, combining quantitative and qualitative survey data. This is part of the national Eldercare project “Enabling medication adherence in the elderly” [8], which focuses on improving medication adherence among older people in Italy. Overall, the Eldercare methodological process includes three stages: (i) a systematic literature review to identify type of adherence‐enhancing interventions in older population (Stage 1) [5]; (ii) a national benchmarking survey of adherence‐related interventions implemented in Italian healthcare settings (Stage 2; Supporting Information 1); (iii) a Strengths, Weaknesses, Opportunities and Threats (SWOT) survey (Stage 3; Supporting Information 2) and qualitative analysis to identify priority areas for action and inform policy makers. Results from the systematic review have been published elsewhere [5] and primarily served to define the conceptual framework and to inform the content domains of the subsequent surveys. Accordingly, the present manuscript focuses on Stages 2 and 3 (Figure 1). Both surveys were administered nationwide, were anonymous and GDPR‐compliant, and accompanied by an electronic information sheet and informed consent. These surveys evaluate perceived implementation and feasibility of adherence‐support practices, not measuring patient‐level adherence rates or clinical outcomes. The overall study protocol received approval from the Bioethics Committee of the Alma Mater Studiorum University of Bologna (protocol nr. 0127857 May 8, 2024).

**FIGURE 1:**
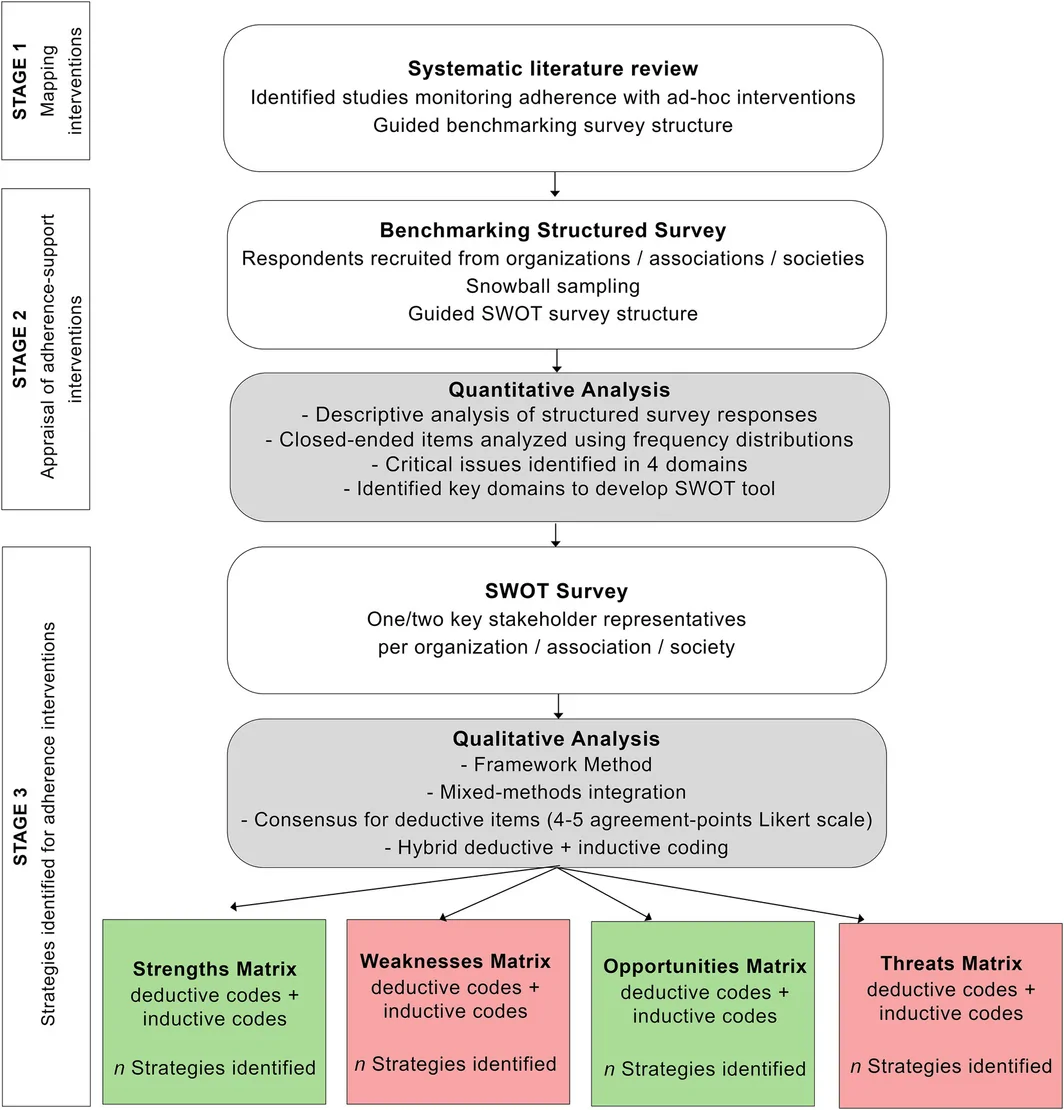
The Eldercare Project process flow.

### 2.2 Sampling Strategy and Participants

Stage 2 consisted of a cross‐sectional online survey delivered via REDCap [9] to physicians, nurses, pharmacists and patient representatives affiliated with Italian scientific societies, professional orders, public healthcare institutions, universities, and patient associations. For this benchmarking survey, we used an organization‐based approach: national organizations were contacted by email and asked to disseminate the survey link to their members (snowball sampling). Because dissemination occurred through participating organizations, the respondent mix reflects organizational reach and engagement, with some settings/professional groups less represented than others. Data were collected between May and August 2024. The questionnaire included five sections with 5‐point Likert items and optional free‐text fields: (i) respondent profile and care setting; (ii) adherence management and local initiatives; (iii) adherence monitoring, including routinely available data sources and documentation tools; (iv) adherence‐improvement interventions, including current implementation and perceived applicability; and (v) unmet needs and barriers. For Stage 3, all organizations participating in Stage 2 were invited to nominate one or two medication‐adherence experts to complete the SWOT survey. For each participating organization, one or two key informants were invited to complete a second REDCap survey between January and March 2025. This nested survey followed the four SWOT domains (strengths, weaknesses, opportunities, threats). Within each domain, we included closed Likert items derived deductively from the main themes emerging in Stage 2 (e.g., communication, coordination, technology use, and financial resources), plus one open‐ended question to capture additional factors. This design provided quantitative estimates of agreement for each statement and complementary qualitative free‐text insights.

### 2.3 Quantitative Analysis

We summarized benchmarking data using descriptive statistics. Items are reported as counts and percentages, with denominators based on non‐missing responses; missing data were not imputed. We described results overall and, where informative, stratified by macro‐area (Northern, Central, Southern Italy) and broad care setting (hospital, primary/territorial care, pharmacy, academic/other). For 5‐point Likert‐scale items (1 = *strongly disagree*, 2 = *disagree*, 3 = *neither agree nor disagree*, 4 = *agree* and 5 = *strongly agree*) responses were treated as ordinal and reported the distribution across categories. In line with scientific literature [10], 4–5 Likert values were considered as agreement with the statement. For the benchmarking survey, Likert results were used primarily in a descriptive way to: (i) quantify the perceived importance and applicability of different tools (e.g., data sources, documentation systems, monitoring approaches), and (ii) identify which interventions to support adherence were judged as more relevant and feasible by healthcare professionals and patient representatives. Data analysis of the five sections was carried out stratifying results by professional role. As an exploratory subgroup analysis, we assessed whether endorsement of unmet needs and barriers differed by Italian macro‐area and by care setting. We compared proportions using *χ* ^2^ tests (or Fisher's exact test when expected counts were < 5) and reported Cramér's V as an effect‐size measure of subgroup differences. Analyses were performed in R (R Foundation for Statistical Computing, Vienna, Austria). Findings from the benchmarking survey were used to derive the structured SWOT statements for Stage 3.

### 2.4 Qualitative Analysis

We analyzed SWOT free‐text responses using the Framework Method described by Gale et al. (2013) [11], following the Standards for Reporting Qualitative Research (SRQR) guidance [12], with a hybrid deductive‐inductive approach [11]. Analyses were intended to describe care‐delivery patterns and implementation gaps to inform quality improvement. The SWOT analysis was intended to prioritize stakeholder‐perceived implementation factors. First, we calculated the proportion of respondents scoring 4–5 in the Likert scale for each structured statement. Statements reaching ≥ 50% agreement were translated into deductive codes. We defined moderate consensus as ≥ 50% and strong consensus as ≥ 75% [13]. Second, we coded open‐ended responses inductively within each SWOT domain to capture additional themes not covered by the structured items. Two researchers coded independently, and discrepancies were resolved through discussion with the co‐authors. Deductive (DC) and inductive (ID) codes were then synthesized into thematic matrices for each SWOT quadrant, and we reported supporting responses as *n*/*N* (%) in summary tables. Finally, themes were organized across macro (policy, regulation, funding), meso (organizational and team level), and micro (direct patient care) levels.

## 3 Results

### 3.1 Benchmarking Survey

These findings reflect respondents' reported practices and perceptions of feasibility in routine care. Of the 32 invited organizations, 56.3% participated (Table S1). In total, 340 respondents completed the survey, representing all Italian regions and a range of professional roles and care settings (Table 1; Figure S1). Most participants were nurses (58.8%), followed by pharmacists (17.9%) and physicians (15.9%). More than half of respondents worked in hospital settings (56.5%), while 17.9% operated in primary/territorial care, 17.9% in pharmacies (Table 1). In the adherence management section, only a limited number of routine practices reached the ≥ 50% endorsement threshold (Figure 2A; Table S2). Adherence appears to be handled mainly during consultations (78.5%). Direct patient‐provider communication was the most frequently reported strategy (51.5% of respondents). This was more frequently reported by physicians' than by other professionals (*p* < 0.05). General practitioners, nurses and clinicians/specialists were perceived as the professionals most involved in managing adherence (75%, 70%, 60%, respectively). Regarding adherence monitoring (Figure 2B; Table S3), 55.3% of respondents reported access to clinical data to monitor adherence. Access to administrative data (dispensing/prescription records), patient self‐reported data, or data derived from digital tools was less frequent and generally remained close to or below 50%. Respondents consistently rated patient‐centered strategies as highly important (Figure 2C; Table S4), including caregiver involvement (94.4%), shared goal setting (90.3%) and individual counseling (87.6%). However, feasibility ratings for implementing the same strategies in local practice were systematically lower. Differences by professional role were observed for selected items (*p* < 0.05), with nurses and pharmacists more supportive than physicians of structured follow‐up and digital monitoring approaches. In the barriers section, limited patient awareness (68.8%) and poor coordination among healthcare professionals (60.3%) emerged as the most frequently reported unmet needs (Figure 2D; Table S5). Key barriers were related to therapeutic complexity and polypharmacy (69.1%), lack of clear information on prescribed therapies (64.7%), insufficient caregiver support (61.5%), and limited resources dedicated to adherence programs. Exploratory subgroup analyses showed modest differences in selected unmet needs and barriers across Italian macro‐areas and care settings (Tables S6 and S7).

**TABLE 1:** Participants to the benchmarking survey (*n* = 340) and SWOT survey (*n* = 14).

Participants' characteristics	Benchmarking survey	SWOT survey
Overall participants, *n*	340	14
Italian regions, *n* (%)		
Northern region	212 (62.4)	5 (35.7)
Southern region	70 (20.6)	8 (57.1)
Central region	58 (17.1)	1 (7.1)
Setting^a^, *n* (%)		
Hospital	192 (56.5)	6 (42.9)
Pharmacy	61 (17.9)	1 (7.1)
Primary/territorial care	61 (17.9)	1 (7.1)
Other	19 (5.6)	1 (7.1)
Academic	7 (2.1)	5 (35.7)
Affiliated organization^b^, *n* (%)		
Professional order	205 (60.3)	1 (7.1)
Scientific society	57 (16.8)	6 (42.9)
Public Healthcare Institution	57 (16.8)	4 (28.6)
Patient association	15 (4.4)	1 (7.1)
Other	4 (1.2)	—
Academia	2 (0.6)	6 (42.9)
Professional role, *n* (%)		
Nurse	200 (58.8)	2 (14.3)
Pharmacist	61 (17.9)	1 (7.1)
Clinician	54 (15.9)	5 (37.1)
Patient representative	16 (4.7)	1 (7.1)
Researcher/educator	5 (1.5)	5 (37.1)
Other	4 (1.2)	—

^a^ Hospital: Hospitals, LHUs hospital, specialist departments; Primary/territorial care: GPs, home care services, community medicine; Pharmacy: Local pharmacies, hospital pharmacies, pharmaceutical services; Academic: Universities, regulatory bodies; Other: scientific societies, academies of scholars, different occupations, private individuals or non‐healthcare professionals (patients).

^b^ More than one answer possible.

**FIGURE 2:**
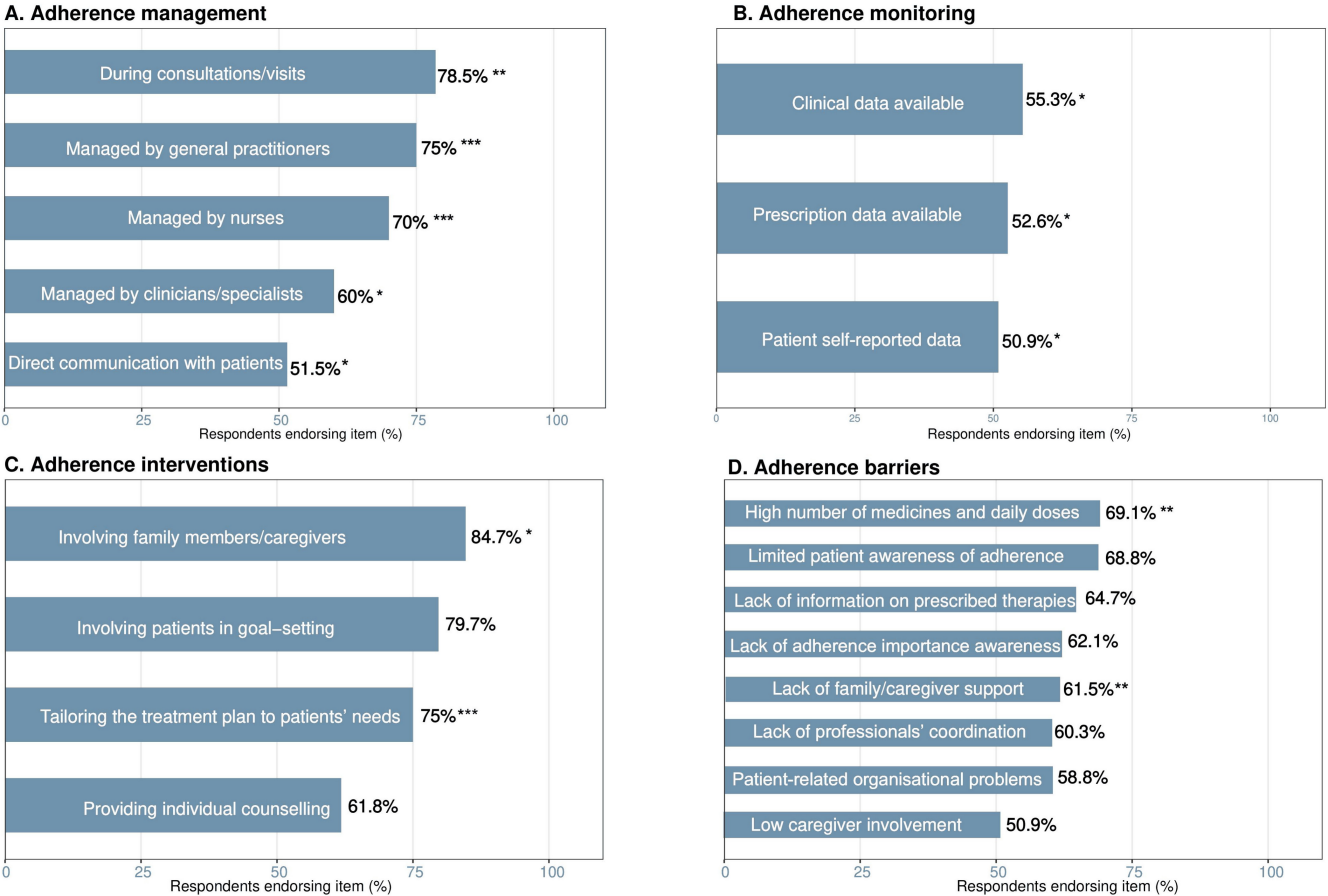
Summary of benchmarking survey on medication adherence management, monitoring, interventions, and barriers in older adults. Multi‐panel bar plot showing the proportion of respondents endorsing each item (Likert scores 4–5) with agreement ≥ 50% in the four benchmarking domains (A: Adherence management applied; B: Adherence monitoring performed; C: Adherence interventions with feasibility; D: Adherence barriers and unmet need found). Bars represent percentages of all respondents; *p < 0.05 for differences across professional roles (*χ* ^2^/Fishers exact test), while **p < 0.01 and ***p < 0.001.

### 3.2 SWOT Analysis

Of 18 invited organizations, 33.3% participated (i.e., 6 organizations; Table S1), each nominating at least one medication‐adherence expert. Fourteen experts completed the survey (Table 1), mainly from Southern Italy (57.1%), followed by Northern (35.7%) and Central regions (7.1%) (Table 1; Figure S1). Participants were primarily researchers/educators (37.1%) and clinicians (37.1%), followed by nurses (14.3%); they were affiliated with academic institutions (42.9%), scientific societies (42.9%) and public healthcare institutions (28.6%). Most worked in hospital settings (42.9%) or academia (35.7%) (Table 1). The survey included 28 closed‐ended statements derived from Stage 2: six strengths, eight weaknesses, eight opportunities and six threats (Table S8). Agreement (Likert 4–5) was reached for 1/6 strengths, 6/8 weaknesses, 8/8 opportunities and 5/6 threats (Table S8), generating 20 deductive codes. Inductive coding of the four open‐ended questions produced additional codes (strengths six, weaknesses six, opportunities four, threats four) (Table S9).

#### 3.2.1 Strengths

Three thematic matrices were identified (Figure 3; Table S9). At the meso level, the main strength was “multiprofessional roles and coordination,” based on one deductive and two inductive codes, and including nurse‐led coordination and collaboration with clinical pharmacology units and trainees. At the micro level, two strengths emerged: “communication and relationship with patient,” capturing direct communication and continuity of the therapeutic relationship, and “education and monitoring,” including clinical/therapeutic monitoring and educational activities.

**FIGURE 3:**
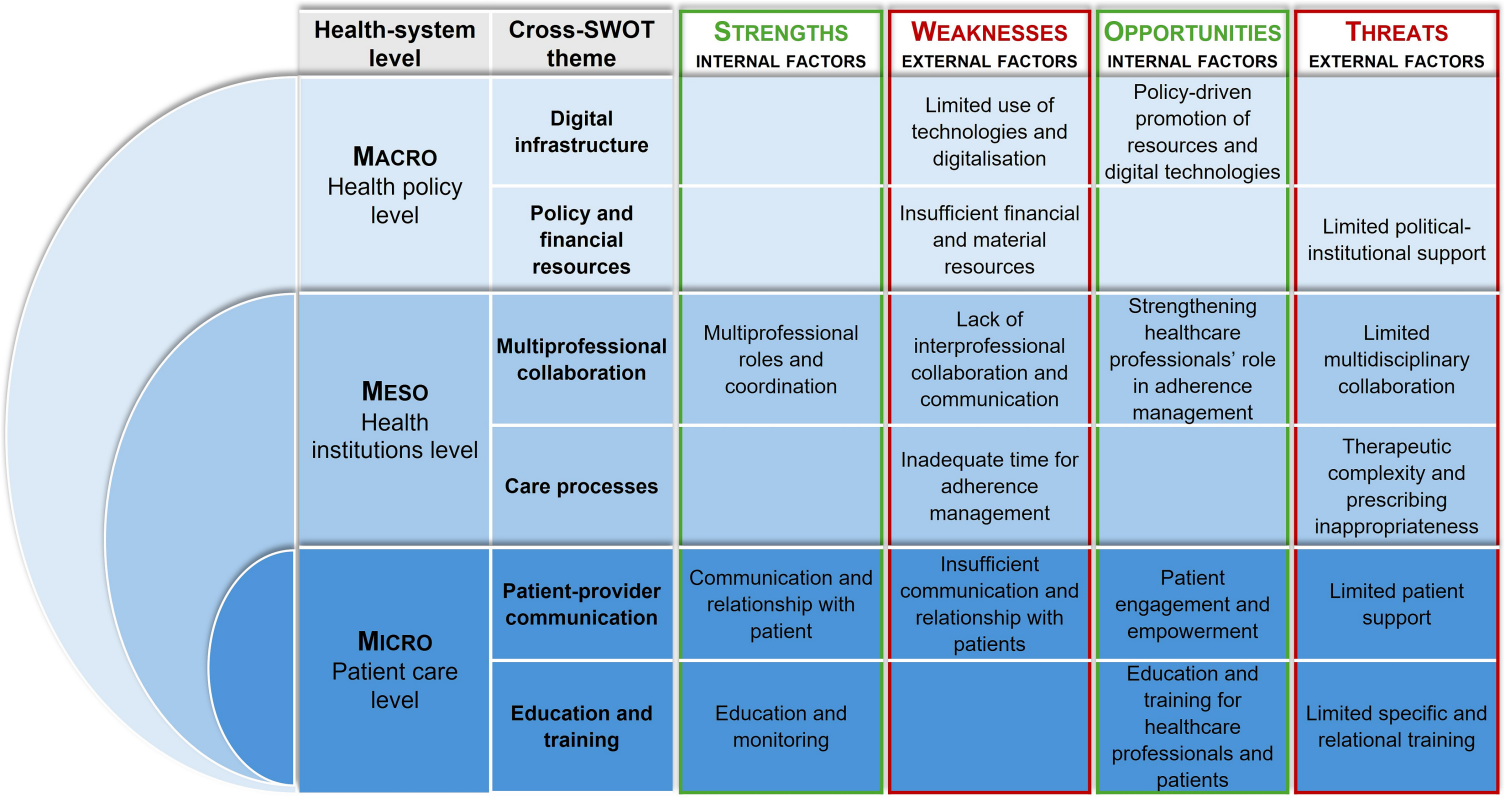
Strategies for the implementation of adherence management interventions in older adults.

#### 3.2.2 Weaknesses

Five thematic matrices were identified (Figure 3; Table S9). At the macro level, “digital infrastructure” reflected the limited use of technologies and digitalisation for adherence monitoring; “insufficient financial and material resources” captured limited access to medicines, scarce funding for adherence programs, lack of professional incentives, and shortages of monitoring tools. At the meso level, “lack of interprofessional collaboration and communication” described poor coordination along the care pathway, training gaps, and weak networking between services; “inadequate time for appropriate adherence management” reflected workload pressure, limited consultation time, and short hospital stays that restrict adherence counseling. At the micro level, “insufficient communication and relationship with patients” mainly reflected limited patient‐provider communication.

#### 3.2.3 Opportunities

Four thematic matrices were identified (Figure 3; Table S9). At the macro level, “policy‐driven promotion of resources and digital technologies” highlighted opportunities to expand digital tools, secure dedicated funding, and strengthen regional policies supporting adherence. At the meso level, “strengthening healthcare professionals' role in adherence management” emphasized an expanded role for general practitioners and nurses within an interprofessional framework. At the micro level, two opportunities emerged: “education and training for healthcare professionals and patients,” addressing continuous professional training and structured patient education, and “patient engagement and empowerment,” including caregiver involvement and stronger collaboration with patient associations.

#### 3.2.4 Threats

Five thematic matrices were identified (Figure 3; Table S9). At the macro level, “limited political‐institutional support” captured insufficient resources for adherence program, limited continuity of local policies and broader lack of institutional commitment. At the meso level, “therapeutic complexity and prescribing inappropriateness” highlighted regimen complexity, polypharmacy and inappropriate prescribing, while “limited multidisciplinary collaboration” reflected fragmented teamwork in routine practice. At the micro level, “limited specific and relational training” indicated gaps in relational skills among healthcare professionals, and “limited patient support” captured low patient awareness and limited availability of psychological support and counseling services.

## 4 Discussion

To the best of our knowledge, this is the first national models‐of‐care quality‐improvement assessment of medication‐adherence interventions for older adults. This study aimed to appraise their perceived quality and feasibility, identifying opportunities for scale‐up across healthcare settings. Within our respondent sample, adherence support remains largely embedded within routine consultations and relies primarily on direct patient–provider communication, with physicians, nurses, and pharmacists as the main actors.

Dedicated organizational structures and digital tools for adherence monitoring remain limited. Strengths included continuity of the therapeutic relationship and nurse‐led coordination while weaknesses and threats centered on fragmented interprofessional collaboration, limited training and digitalisation, and scarce financial and institutional support for structured programs. These findings align with international evidence showing that medication adherence in older adults reflects patient‐, provider‐ and system‐level determinants and that routine care still relies largely on counseling and clinical follow‐up rather than structured, reimbursed services [14, 15].

Our results reinforce the need to move beyond individual clinical encounters toward more coordinated and system‐level approaches to adherence management.

### 4.1 Lessons Learned and Opportunities for Improvement: Systems and Reimbursement Models

A key threat emerging from the Italian SWOT analysis is the lack of specific financial incentives and dedicated resources for adherence‐improvement programs. This may partly reflect Italy's decentralized National Health Service, where regions are responsible for organizing and delivering care, potentially leading to heterogeneous prioritization of adherence‐oriented services [16]. More broadly, adherence programs often require upfront investment in training, workforce time, technology and monitoring infrastructure, while benefits accrue over longer time horizons. Similar challenges have been reported across European health systems [17]. Some countries have nevertheless introduced structured services to support adherence. Belgium, Denmark, Finland, the Netherlands and the United Kingdom (UK), for example, have implemented reimbursement schemes for services including multidose drug dispensing (MDD) [18] and structured medication reviews [17]. In the UK, community pharmacy services such as the New Medicine Service provide early counseling and follow‐up for patients starting new treatments within a national policy framework informed by NICE guidance [19, 20, 21, 22]. Emerging organizational models also illustrate how adherence management can be strengthened through interprofessional collaboration. For example, Slovenia has integrated clinical pharmacists into primary care teams to conduct medication reviews for patients with complex treatment regimens [23]. These experiences suggest that health systems investing in structured services and clearly defined professional roles may be better positioned to implement coordinated adherence‐management pathways [23].

In Italy, adherence management remains centered on general practitioners, specialists, and pharmacists, but multidisciplinary collaboration is not yet systematic. Overall, health systems differences likely reflect how services are commissioned and funded, how professional roles are formalized, and whether shared data infrastructures and interprofessional workflows are integrated in routine care. This international comparison may contextualize implementation levers rather than imply direct cross‐country comparability, particularly with heterogeneous systems such as the United States.

### 4.2 System‐ and Patient‐Level Barriers

The most frequently reported barriers to adherence in Italy are constraints on clinicians' time and limited patient awareness of the importance of taking medicines as prescribed, in line with most European countries [14, 23]. In many healthcare systems, adherence support is expected to be delivered during routine consultations, where limited consultation time and competing clinical priorities may constrain systematic assessment and counseling [14]. In the present study, these barriers are reflected in the central role of therapeutic complexity (polypharmacy and regimen complexity), together with limited information and coordination among professionals. Structural barriers also vary across contexts: in low‐ and middle‐income settings, constraints may include medication costs or supply shortages [24]. In contrast, in Italy, economic access and drug availability were less frequently cited, possibly reflecting universal coverage and reimbursement for medicines, although cost sharing and regional differences can still affect access for some groups [25]. Among uninsured, low‐income older adults in the United States, out‐of‐pocket costs and transport difficulties to reach pharmacies are among the strongest predictors of non‐adherence [26]. Psychosocial determinants such as stress, depression and family dynamics may also influence adherence behaviors but remain under‐recognized in routine clinical practice [27].

### 4.3 Use of Technologies and Data for Adherence Monitoring

The use of digital tools and routinely collected data to monitor adherence varies widely across health systems, In Northern Europe, the Netherlands and the UK, and in some non‐European settings such as the United States, South Korea and China, digital solutions such as apps, telemonitoring, smart blister packs, SMS reminders are more commonly used, often alongside linked clinical and administrative data to support risk stratification and follow‐up [21, 22, 28, 29, 30, 31, 32, 33]. In Italy, adherence monitoring relies mainly on clinical information and prescription or dispensing data, while dedicated digital tools remain limited. This may reflect both patient‐level barriers, such as digital literacy among older adults [34, 35], and system‐level factors such as regional disparities in digital infrastructure [25]. This pattern is consistent with European data indicating that only about 10%–15% of healthcare professionals regularly use technological solutions to improve adherence [36]. The Netherlands represents a benchmark for automated dose‐dispensing devices with resulting in improvements in adherence from 49% to 97% after their introduction [21]. Albeit this, organizational barriers are still declared (i.e., healthcare professionals workload and resource issues) [21]. In the United States, older adults rely largely on simple pill organizers and less in mobile health apps [28]. Overall, international evidence suggests that technology alone is unlikely to substantially improve adherence unless it is embedded within coordinated care pathways including medication review, counseling and structured follow‐up.

### 4.4 Strengths and Limitations

This study provides the first national mapping of medication‐adherence management in older adults in Italy, combining perspectives from scientific societies, professional orders, public healthcare institutions and patient associations. The mixed‐method design integrates quantitative benchmarking with qualitative framework analysis and includes multiple professional roles and care settings. Because data were self‐reported, they may not reflect actual delivered actions in all settings. Findings should therefore be interpreted as implementation and feasibility evidence to inform models of care and quality improvement, rather than as estimates of adherence outcomes. Limitations include voluntary organizational participation, which may have favored stakeholders more engaged with adherence initiatives. Compared with national population distribution in 2024 (North ~46.6%, Centre ~19.9%, South ~33.5%), the benchmarking sample overrepresented Northern regions (62.4%) and underrepresented Southern (20.6%) [37]. This reflects our organization‐based dissemination approach and supports interpreting results as stakeholder‐reported implementation rather than a nationally representative estimate. The SWOT survey included *n* = 14 experts, and non‐response may have introduced selection bias. Due to this sample size, exploratory subgroup comparisons were not performed for the SWOT analysis. Therefore, SWOT findings should be interpreted as priorities identified by participating key informants, used to complement the broader national survey. Findings should therefore be interpreted as perceptions rather than measured performance. Samples were limited and uneven across professions, with nurses over‐represented, which may have amplified nurse‐led activities, although this aligns with their central role in older patients' care and evidence supporting nurse‐led adherence support in chronic conditions [38]. As a result, some findings may reflect hospital‐ and nursing‐led workflows more than office‐based outpatient practice. We therefore interpret results as stakeholder‐reported implementation and feasibility rather than a fully representative description of all care settings. Data were self‐reported and cross‐sectional, and awareness of local initiatives may have varied across respondents. Finally, the focus on Italy may limit generalizability to other health systems and to specific subgroups of older adults. Because Italy has a universal National Health Service, our findings may not be directly generalizable to US geriatric practice, where financing, access, and care delivery are more heterogeneous. However, the implementation framework and identified levers (workflows, roles, incentives, and data infrastructure) are intended to be transferable and adaptable across systems. Despite these limitations, the study offers a robust basis to inform national recommendations and guide future implementation initiatives.

Taken together, these findings suggest that improving medication adherence in older adults requires moving beyond consultation‐based approaches toward coordinated interprofessional care pathways supported by organizational resources, professional training, and better use of health data.

## 5 Conclusion

Medication‐adherence support for older adults in Italy remains largely embedded within routine consultations and lacks structured organizational support. Strengthening interprofessional coordination, workforce training, and integration of digital and administrative data may help aging health systems develop a more structured and scalable quality‐improvement model of care to support adherence among older adults.

## Author Contributions

All authors meet the criteria for authorship stated in the Uniform Requirements for Manuscripts Submitted to Biomedical Journals. Conceptualization: E.M. and A.N. Methodology: S.M., L.P., E.M., and A.N. Software: S.M. and L.P. Validation: E.M. and A.N. Data curation: S.M. and L.P. Writing – original draft preparation: S.M. and L.P. Writing – review and editing: M.C., M.D., C.L., E.O., V.O., E.R., S.S., M.S., E.P., A.N., and E.M. Supervision: E.M. and A.N. Funding acquisition: E.M. All authors have read and agreed to the published version of the manuscript.

## Funding

Eldercare (Enabling Medication Adherence in the Elderly) project is a research project aimed at collecting and documenting the most relevant experiences developed in Italy at the national, regional, and local levels to enhance medication adherence in the elderly. The project is funded by the Italian Ministry of University and Research‐ European Union‐Next Generation EU (PRIN—Call 2022 Prot. 20227C2YLA) and European Medicine Agency (Tender N. EMA/2020/46/TDA/30Lot4). The funders had no role in the study design, data collection, management, analysis, or interpretation, manuscript preparation, or the decision to submit the manuscript for publication. The views expressed are solely those of the authors.

## Conflicts of Interest

The authors declare no conflicts of interest.

## Supporting information


**Supporting Information 1:** Benchmarking survey instrument (Stage 2): national survey on medication‐adherence management, monitoring, interventions, and barriers in older adults.
**Supporting Information 2:** SWOT survey instrument (Stage 3): stakeholder assessment of strengths, weaknesses, opportunities, and threats for medication‐adherence interventions in older adults.
**Supporting Information 3:** Supplementary figures and tables: response rates, geographic distribution, detailed benchmarking results, SWOT item agreement, coding matrices, and exploratory subgroup analyses by Italian macro‐area and care setting.
**Figure S1:** Geographical distribution of participants to the benchmarking survey (*n* = 343) and SWOT survey (*n* = 14).
**Table S1:** Response rates to the benchmarking survey and SWOT survey.
**Table S2:** Adherence management according to benchmarking survey participants (survey section 2).
**Table S3:** Adherence monitoring according to benchmarking survey participants (survey section 3).
**Table S4:** Adherence interventions according to benchmarking survey participants (survey section 4).
**Table S5:** Adherence barriers and unmet needs according to benchmarking survey participants (survey section 5).
**Table S6:** Unmet needs and barriers endorsed by benchmarking respondents, stratified by Italian macro‐area (North, Centre, South).
**Table S7:** Unmet needs and barriers endorsed by benchmarking respondents, stratified by care setting.
**Table S8:** Agreement rates and consensus levels for SWOT items (proportion of respondents scoring 4–5 on the 5‐point Likert scale).
**Table S9:** Summary of thematic matrices and generative codes from the SWOT analysis.

## Data Availability

The data generated and analyzed during this study are included in this published article and its Supporting Information in aggregated form.
