# Supplementary material for: Medication Adherence Interventions for Older Adults: A National Mixed‐Methods Study of Implementation Across Italian Healthcare Settings
Source: J Am Geriatr Soc. 2026 Aug 3;74(10):3070–9. doi: 10.1111/jgs.70596 (PMC13723202; doi:10.1111/jgs.70596)
Supplement: Supplementary file 1 [file JGS-74-3070-s001.pdf]

Supplementary files

Medication Adherence Interventions for Older Adults: A National Mixed-Methods Study of Implementation across Italian Healthcare Settings

Supplementary Material 1.

Benchmarking survey instrument (Stage 2): national survey on medication-adherence management, monitoring, interventions, and barriers in older adults.

A Benchmarking Survey on interventions for managing medication adherence in the older adults

Dear Participant,

We are pleased to invite you to participate in a research initiative that aims to collect data on interventions to improve medication adherence in Italy.

Medication adherence is a complex challenge influenced by various factors, including healthcare infrastructure, social dynamics and individual health conditions. Through our commitment to researching and developing effective strategies to improve treatment adherence, we are seeking to identify the best practices already implemented in various healthcare settings in Italy.

Your experience and perspective are crucial to the success of this research. The aim of this questionnaire is to gather detailed information on the professionals and organisations involved in medication adherence, as well as the practices and interventions currently in place.

This research activity is part of a larger project funded by the European Union - Next Generation EU (PRIN - Call 2022 Prot. 20227C2YLA). More details about the project are available at the following link.

We kindly ask you to spend about 10 minutes of your time to complete the survey. Your contribution will help us create a comprehensive overview of the current therapy adherence initiatives already in place in Italy.

We appreciate your contribution to this important initiative!

Thank you in advance for your cooperation. If you have any questions or concerns, please do not hesitate to contact us.

DATA MANAGEMENT INFORMATION NOTICE
Security measures will be implemented to protect the confidentiality of your data. You will not be asked to provide any information that could identify you or link your responses to you (e.g., name or email address), and IP addresses will not be tracked. The information you provide will be processed electronically by specifically authorized personnel, in accordance with applicable data protection legislation (ex-Article 13 of Regulation (EU) 2016/679-GDPR). At the end of the study, research findings will be disseminated only in aggregate form, through scientific publications and/or presentations at meetings, conferences, congresses, and other scientific and public engagement events.
By submitting the questionnaire, you declare that you have read and understood the statements above.

Section 1 (S1) – Participant general information			
**Item ID**	**Item wording***	**Response format**	**Response options**
**S1-Q1**	Please indicate your region	Single choice (drop-down)	Italian regions (drop-down list)
**S1-Q2**	Please indicate your professional role(s). (Select all that apply)	Multiple choice	*Scientific societies:* Academic; Clinician; Scientific society representative; Scientific federations representative.<br><br>*Professional orders*: Director of Pharmaceutical Policies; Director General (Health); Regional executives/managers; Professional order representative.<br><br>*Patient associations:* Patient association representative.<br><br>*Other health professions*: General practitioner; Nurse; Hospital or community pharmacist; Community pharmacist.<br><br>Other (please specify): ____
**S1-Q3**	Which type(s) of organization do you represent? (Select all that apply)	Multiple choice	Hospital;<br>Primary care;<br>National agency for regional health services;<br>Local and regional health directorates;<br>Research/University;<br>Community or hospital pharmacy;<br>Private organization/Company;<br>Government body; Consulting company;<br>Scientific society/association (please specify): ____;<br>Scientific federations (please specify): ____;<br>Patient association (please specify): ____;<br>Professional orders (please specify): ____;<br>Other (please specify): ____

*Mandatory fields

Section 2 (S2) – Medication adherence: management			
**Item ID**	**Item wording***	**Response format**	**Response options**
**S2-Q1**	Is medication adherence assessed in your care setting?	Single choice	Yes;<br>No;<br>Not sure
**S2-Q2**	If yes, which tool(s) are used to assess medication adherence? (Select all that apply)	Multiple choice	Direct patient interview/communication;<br>Patient questionnaires;<br>Caregiver questionnaires;<br>Physician questionnaires;<br>Monitoring of prescribing/dispensing data;<br>Mobile applications (apps);<br>Programmed dosing devices (pill box/medication dispenser);<br>Wearable devices;<br>None of the above;<br>Other (please specify): ____
**S2-Q3**	Which professional group(s) are involved in medication adherence management? (Select all that apply)	Multiple choice	General practitioner (GP);<br>Clinicians/Specialists;<br>Clinical pharmacologist;<br>Community/territorial pharmacists;<br>Hospital pharmacists;<br>Nurses;<br>Researchers;<br>Other healthcare staff (e.g., medical technicians, physiotherapists, midwives, nursing assistants, assistants);<br>No professional group involved;<br>Other (please specify): ____
**S2-Q4**	At which point(s) along the therapeutic pathway is medication adherence assessed? (Select all that apply)	Multiple choice	During consultations/visits;<br>At the time of prescribing;<br>At the time of dispensing;<br>Remote consultations;<br>During transitions/transfer between care settings;<br>No method available;<br>Other (please specify): ____

*Mandatory fields

Section 3 – Medication adherence: monitoring			
**Item ID**	**Item wording***	**Response format**	**Response options**
**S3-Q1**	Which data do healthcare professionals in your region have access to regarding medication adherence?<br>(Select all that apply)	Multiple choice	Clinical data;<br>Dispensing data;<br>Prescribing data;<br>Data derived from ad-hoc devices;<br>Patient self-report; Records of visits and follow-up;<br>Data from remote monitoring;<br>Data collected during phone or virtual interactions with the patient;<br>Laboratory test results related to therapy;<br>Adherence reports generated by digital applications;<br>No access to patient-level data;<br>Other (please specify): _____
**S3-Q2**	How do you rate the importance of using the following tools for documenting medication adherence?	Likert scale for each item:<br>1 = Not important at all;<br>2= Not important;<br>3= Neutral;<br>4= Important;<br>5 = Extremely important.	Patient medical record;<br>Inform the patient and/or caregiver;<br>Dedicated app;<br>Ward-specific register;<br>Electronic health record;<br>Healthcare professional follow-up report;<br>Local/regional information systems;<br>Scientific society database;<br>Dedicated online platform;<br>Ward adherence assessment report.

*Mandatory fields

Section 4 (S4) – Medication adherence: interventions			
**Item ID**	**Item wording**	**Response format**	**Response options**
**S4-Q3**	How do you rate the importance of the following interventions to improve medication adherence?*	Likert scale for each item:<br>1 = Not important at all;<br>2= Not important;<br>3= Neutral;<br>4= Important;<br>5 = Extremely important.	Provide individual counselling;<br>Arrange more frequent follow-ups;<br>Refer to specific adherence programs;<br>Use dedicated smartphone apps;<br>Involve the patient in support groups;<br>Tailor therapeutic plan to patient needs;<br>Involve caregivers in adherence;<br>Send reminders via SMS or email;<br>Use remote monitoring tools;<br>Involve patient in defining therapeutic goals.
**S4-Q4**	How do you rate the feasibility and practical implementability of the following interventions to improve medication adherence?*	Likert scale for each item:<br>1 = Not applicable at all;<br>2= Not applicable;<br>3= Neutral;<br>4= Applicable;<br>5 = Extremely applicable.	Provide individual counselling;<br>Arrange more frequent follow-ups;<br>Refer to specific adherence programs;<br>Use dedicated smartphone apps;<br>Involve the patient in support groups;<br>Tailor therapeutic plan to patient needs;<br>Involve caregivers in adherence;<br>Send reminders via SMS or email;<br>Use remote monitoring tools;<br>Involve patient in defining therapeutic goals.
**S4-Q5**	Can you identify one or more national policies, guidelines, best practices, or campaigns on medication adherence-existing and/or planned-in your care setting, your region, or in Italy more broadly, that you are aware of and consider valuable?<br>If available, please provide a website link where they can be accessed, or indicate that you will upload the documents later.*	Open-ended (free text)	Free-text field (with optional URL(s))
**S4-Q6**	Please upload any document(s) related to your previous answer.**	File upload	Upload a document (.pdf .doc)

*Mandatory fields
** Optional fields

Section 5 (S5) – Medication adherence: unmet needs and barriers			
**Item ID**	**Item wording***	**Response format**	**Response options**
**S5-Q7**	What do you identify as an unmet need regarding medication adherence? (Select all that apply)	Multiple choice	Lack of financial resources for support programmes;<br>Limited patient awareness of the importance of adherence;<br>Limited involvement of caregivers in the care process;<br>Need for personalised and flexible interventions;<br>Lack of specific training for healthcare professionals on adherence;<br>Limited access to monitoring technologies and remote support;<br>Absence of structured follow-up programmes;<br>Linguistic and cultural barriers in communication;<br>Need for strategies to manage medication side effects;<br>Lack of coordination among healthcare professionals along the care pathway;<br>Lack of time during patient visits;<br>Other (please specify): ____
**S5-Q8**	Which barriers do you identify as hindering appropriate medication adherence?<br>(Select all that apply)	Multiple choice	Lack of clear information about prescribed therapies;<br>Distrust in medicine and medications;<br>Financial difficulties and limited access to medications;<br>Perceived adverse effects;<br>High number of medications and daily dosing frequency;<br>Patient memory or time-management problems;<br>Lack of family or caregiver support;<br>Linguistic and cultural barriers to understanding instructions;<br>Lack of awareness of the importance of adherence;<br>Limited access to counselling services or psychological support;<br>Limited availability of structured follow-up programmes;<br>Inadequate communication and coordination among healthcare professionals;<br>Other (please specify): ____

*Mandatory fields

Supplementary Material 2.

SWOT survey instrument (Stage 3): stakeholder assessment of strengths, weaknesses, opportunities, and threats for medication-adherence interventions in older adults.

A SWOT survey on interventions for managing medication adherence in the older adults

Dear Colleague,

We are pleased to invite you to participate in the next phase of our research, which aims to analyse the effectiveness of interventions to improve treatment adherence in Italy through a SWOT analysis, i.e. an analysis of the strengths (*Strengths*), weaknesses (*Weaknesses*), opportunities (*Opportunities*) and threats (*Threats*) in relation to the management of medication adherence in your care setting.

After completing the benchmarking survey at national level, which gathered valuable information from professionals across the country, we would now like to involve you in this next phase of the project.

The aim of the SWOT analysis is to examine the quantitative data collected at national level (over 300 responses) and identify the key factors that could influence the success of strategies to improve medication adherence in our country.

Your perspective on the healthcare setting you represent is essential for assessing the strengths, challenges, opportunities, and risks associated with implementing these interventions in the context of the Italian healthcare system.

This research activity is part of a larger project funded by the European Union through Next Generation EU (PRIN - Call 2022 Prot. 20227C2YLA). Further details about the project are available at the following link.

We thank you for your time and valuable contribution, which is essential for shaping the future of treatment adherence practices in Italy.

We thank you in advance for your cooperation. If you have any questions or require further clarification, please do not hesitate to contact us.

DATA MANAGEMENT INFORMATION NOTICE
Security measures will be implemented to protect the confidentiality of your data. You will not be asked to provide any information that could identify you or link your responses to you (e.g., name or email address), and IP addresses will not be tracked. The information you provide will be processed electronically by specifically authorized personnel, in accordance with applicable data protection legislation (ex-Article 13 of Regulation (EU) 2016/679-GDPR). At the end of the study, research findings will be disseminated only in aggregate form, through scientific publications and/or presentations at meetings, conferences, congresses, and other scientific and public engagement events.
By submitting the questionnaire, you declare that you have read and understood the statements above.

**SECTION 1 General Information**	***Open-ended question***
**Please indicate your occupation:**	*open text field*
**Please indicate your reference organisation:**	*open text field*
**Please indicate your region of residence:**	*open text field*

**SECTION 2 SWOT Analysis description**
In this section, we will focus on the main characteristics of managing medication adherence in your care setting, using the SWOT assessment tool. You will be asked to indicate your level of agreement with each of the statements, using a scale from 1 to 5, identifying, based on your experience, the strengths (“S”), weaknesses (“W”), opportunities (“O”) and threats (“T”) in relation to managing medication adherence in your care setting.

Strengths ("S")	5-point Likert-scale				
	strongly disagree	disagree	neither agree nor disagree	agree	strongly agree
The management of medication adherence in my care setting has strong points that distinguish it from other settings.	1	2	3	4	5
The human resources for managing medication adherence (qualified staff) are adequate.	1	2	3	4	5
The direct involvement of healthcare professionals (doctors, nurses, pharmacists) in discussions with patients effectively supports medication adherence in my care setting.	1	2	3	4	5
Good practices are already in place in my care setting.	1	2	3	4	5
There is strong collaboration between the various healthcare professionals.	1	2	3	4	5
Effective technological tools are available for monitoring adherence.	1	2	3	4	5
The availability of clinical and prescription data is sufficient and allows for effective monitoring of medication adherence.	1	2	3	4	5
Here is a list of the main strengths you identify in your care setting:	***open text field***				

Weaknesses "W"	5-point Likert-scale				
	strongly disagree	strongly disagree	strongly disagree	strongly disagree	strongly disagree
The management of medication adherence in my care setting has some weaknesses that could be improved.	1	2	3	4	5
Patients do not receive adequate or understandable information about their prescribed therapies, which negatively affects medication adherence.	1	2	3	4	5
Patients are not sufficiently involved in setting therapeutic goals, which limits their medication adherence.	1	2	3	4	5
Poor coordination between different healthcare professionals throughout the care pathway is a significant obstacle to adherence management.	1	2	3	4	5
The use of wearable devices and digital applications to monitor treatment adherence is still insufficient in my care setting.	1	2	3	4	5
There are gaps in the training of healthcare professionals.	1	2	3	4	5
Access to certain medicines for patients is limited due to factors such as their availability, distribution or the costs associated with the medicines.	1	2	3	4	5
The financial resources allocated to medication adherence support programmes, such as educational campaigns, patient monitoring and healthcare staff training, are insufficient in my care setting.	1	2	3	4	5
There are no incentives for healthcare professionals to improve the management of medication adherence.	1	2	3	4	5
Here is a list of the main weaknesses you have identified in your care setting:	***open text field***				

Opportunities "O"	**5-point Likert-scale**				
	**strongly disagree**	**strongly disagree**	**strongly disagree**	**strongly disagree**	**strongly disagree**
There are many opportunities to improve adherence management.	1	2	3	4	5
Implement new training programmes for healthcare professionals.	1	2	3	4	5
Raising patient awareness of the importance of adherence through educational programmes can lead to significant improvements.	1	2	3	4	5
Establish partnerships with patient organisations/associations to raise awareness.	1	2	3	4	5
Increasing the role of nurses and general practitioners in monitoring medication adherence could significantly improve adherence management.	1	2	3	4	5
Involve family members or caregivers in setting and monitoring therapeutic goals.	1	2	3	4	5
Encourage the use of digital technologies for monitoring adherence.	1	2	3	4	5
Encourage access to specific funding to develop and implement interventions aimed at improving treatment adherence (such as monitoring programmes, patient support and training for healthcare professionals).	1	2	3	4	5
Implement improvement strategies in regional health policies.	1	2	3	4	5
Here is a list of the main opportunities you identify in your care setting:	***open text field***				

Threats "T"	**5-point Likert-scale**				
	**strongly disagree**	**strongly disagree**	**strongly disagree**	**strongly disagree**	**strongly disagree**
There are some critical issues that could negatively affect the adherence management in my care setting.	1	2	3	4	5
The high number of medications and the complexity of treatment regimens are an obstacle to medication adherence for patients.	1	2	3	4	5
Patients have limited awareness of the importance of following treatments correctly, which hinders the success of adherence interventions.	1	2	3	4	5
The limited availability of psychological support and counselling services is a barrier to patients maintaining medication adherence.	1	2	3	4	5
Financial resources for medication adherence programmes are scarce, limiting the possibility of implementing effective interventions.	1	2	3	4	5
Frequent changes in regional health policies hinder the continuity and effectiveness of interventions to improve medication adherence.	1	2	3	4	5
Lack of support from local institutions.	1	2	3	4	5
Here is a list of the main critical issues identified in your care setting:	***open text field***				

Supplementary Material 3.

Supplementary figures and tables: response rates, geographic distribution, detailed benchmarking results, SWOT item agreement, coding matrices, and exploratory subgroup analyses by Italian macro-area and care setting.

Supplementary material 3

Figures and Tables Legend

Figure S1. Geographical distribution of participants to the benchmarking survey (n=343) and SWOT survey (n=14).

Table S1. Response rates to the benchmarking survey and SWOT survey.

Table S2. Adherence management according to benchmarking survey participants (survey section 2).

Table S3. Adherence monitoring according to benchmarking survey participants (survey section 3).

Table S4. Adherence interventions according to benchmarking survey participants (survey section 4).

Table S5. Adherence barriers and unmet needs according to benchmarking survey participants (survey section 5).

Table S6. Unmet needs and barriers endorsed by benchmarking respondents, stratified by Italian macro-area (North, Centre, South).

Table S7. Unmet needs and barriers endorsed by benchmarking respondents, stratified by care setting.

Table S8. Agreement rates and consensus levels for SWOT items (proportion of respondents scoring 4–5 on the 5-point Likert scale).

Table S9. Summary of thematic matrices and generative codes from the SWOT analysis.

Figure S1. Geographical distribution of participants to the benchmarking survey (n=340) and SWOT survey (n=14).

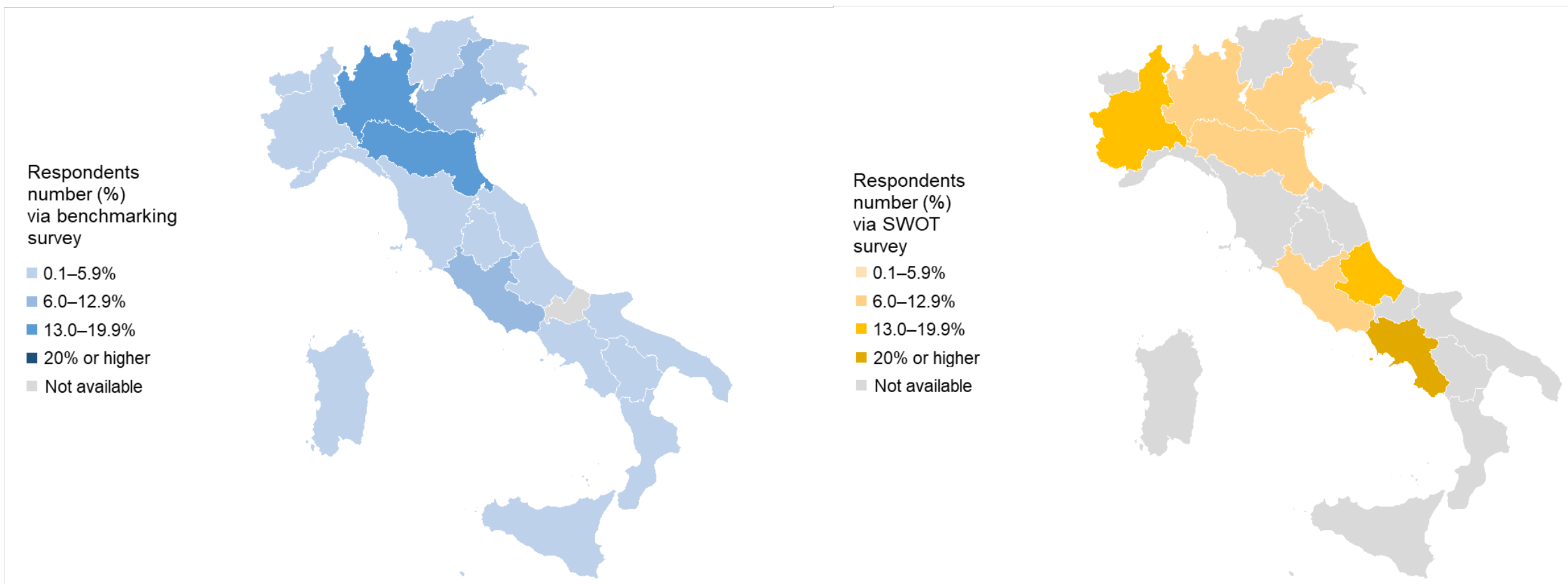

Table S1. Response rates to the benchmarking survey and SWOT survey.

**Surveys**	**Invited organisations *(n)***	**Participant organisations *(n)***	**Response rate *(%)***
**Benchmarking survey**	**32**	**18**	**56.3**
Scientific societies	20	12	60.0
Professional orders	4	3	75.0
Patient associations	4	3	75.0
Geriatric associations	4	0	-
**SWOT survey**	**18**	**6**	**33.3**
Scientific societies	12	4	33.3
Professional orders	3	1	33.3
Patient associations	3	1	33.3

Table S2. Adherence management according to benchmarking survey participants (survey section 2).

**Section on adherence management**	**Overall n (%)**	**Nurse n (%)**	**Pharmacist n (%)**	**Clinician n (%)**	**Patient representativ en (%)**	**Researcher /Educator n (%)**	**Other n (%)**	***p-value***
**Respondents**	**340**	**200 (58.8)**	**61 (17.9)**	**54 (15.9)**	**16 (4.7)**	**5 (1.5)**	**4 (1.2)**	-
**Tools used to handling adherence**								
Direct communication with patients	175 (51.5)	100 (50.0)	25 (41.0)	39 (72.2)	6 (37.5)	3 (60.0)	2 (50.0)	0.019
Monitoring of prescription/drug dispensing data	79 (23.2)	42 (21.0)	28 (45.9)	3 (5.6)	3 (18.8)	1 (20.0)	2 (50.0)	<0.001
Patient questionnaires	36 (10.6)	19 (9.5)	10 (16.4)	4 (7.4)	3 (18.8)	-	-	0.393
Caregiver questionnaires	31 (9.1)	18 (9.0)	3 (4.9)	8 (14.8)	1 (6.3)	-	1 (25.0)	0.381
Scheduled administration devices (pill boxes or drug dispensers)	24 (7.1)	18 (9.0)	3 (4.9)	3 (5.6)	-	-	-	0.599
Mobile applications	12 (3.5)	9 (4.5)	3 (4.9)	-	-	-	-	0.580
Clinician questionnaires	6 (1.8)	5 (2.5)	1 (1.6)	-	-	-	-	0.842
Wearable devices	4 (1.2)	3 (1.5)	1 (1.6)	-	-	-	-	0.942
**Professions involved in adherence management**								
General practitioners	255 (75.0)	145 (72.5)	43 (70.5)	52 (96.3)	6 (37.5)	5 (100)	4 (100)	<0.001
Nurses	238 (70.0)	185 (92.5)	17 (27.9)	28 (51.9)	4 (25.0)	2 (40.0)	2 (50.0)	<0.001
Clinicians/Specialists	204 (60.0)	112 (56.0)	34 (55.7)	41 (75.9)	13 (81.3)	3 (60.0)	1 (25.0)	0.028
Community pharmacists	96 (28.2)	33 (16.5)	42 (68.9)	15 (27.8)	2 (12.5)	4 (80.0)	-	<0.001
Hospital pharmacists	80 (23.5)	27 (13.5)	45 (73.8)	5 (9.3)	1 (6.3)	1 (20.0)	1 (25.0)	<0.001
Other (technicians, physiotherapists, assistants)	46 (13.5)	33 (16.5)	3 (4.9)	3 (5.6)	4 (25.0)	1 (20.0)	2 (50.0)	0.011
Clinical pharmacologists	42 (12.4)	17 (8.5)	16 (26.2)	6 (11.1)	2 (12.5)	-	1 (25.0)	0.011
Researchers	23 (6.8)	10 (5.0)	4 (6.6)	3 (5.6)	5 (31.3)	1 (20.0)	-	0.003
**Time points of adherence assessment**								
During consultations/visits	267 (78.5)	160 (80.0)	38 (62.3)	49 (90.7)	12 (75.0)	5 (100.0)	3 (75.0)	0.007
During transfer between care settings	162 (47.6)	102 (51.0)	30 (49.2)	20 (37.0)	4 (25.0)	4 (80.0)	2 (50.0)	0.118
At dispensing	133 (39.1)	66 (33.0)	53 (86.9)	8 (14.8)	5 (31.3)	1 (20.0)	-	<0.001
At prescription	107 (31.5)	66 (33.0)	20 (32.8)	15 (27.8)	2 (12.5)	2 (40.0)	2 (50.0)	0.538
Remote consultations	59 (17.4)	39 (19.5)	9 (14.8)	5 (9.3)	4 (25.0)	2 (40.0)	-	0.246

*Other includes professionals in non-clinical roles (e.g. healthcare managers, freelance experts affiliated with scientific societies).

Notes: p-value less than 0.05 was considered statistically significant.

Table S3. Adherence monitoring according to benchmarking survey participants (survey section 3).

**Section on adherence monitoring**	**Overall n (%)**	**Nurse n (%)**	**Pharmacist n (%)**	**Clinician n (%)**	**Patient representativen (%)**	**Researcher/ Educator n (%)**	**Other n (%)**	***p-value***
**Respondents**	**340**	**200 (58.8)**	**61 (17.9)**	**54 (15.9)**	**16 (4.7)**	**5 (1.5)**	**4 (1.2)**	-
**Type of adherence-related data available**								
Clinical data	188 (55.3)	123 (61.5)	24 (39.3)	28 (51.9)	10 (62.5)	1 (20.0)	2 (50.0)	0.028
Prescription data	179 (52.6)	101 (50.5)	43 (70.5)	22 (40.7)	9 (56.3)	3 (60.0)	1 (25.0)	0.027
Patient self-reported data	173 (50.9)	104 (52.0)	21 (34.4)	37 (68.5)	7 (43.8)	2 (40.0)	2 (50.0)	0.016
Remote monitoring data	128 (37.6)	77 (38.5)	17 (27.9)	23 (42.6)	5 (31.3)	3 (60.0)	3 (75.0)	0.234
Dispensing data	93 (27.4)	33 (16.5)	46 (75.4)	12 (22.2)	1 (6.3)	1 (20.0)	-	<0.001
Laboratory test results related to therapies	85 (25.0)	56 (28.0)	11 (18.0)	12 (22.2)	3 (18.8)	2 (40.0)	1 (25.0)	0.594
Data from telephone or virtual interactions with patients	66 (19.4)	45 (22.5)	11 (18.0)	8 (14.8)	2 (12.5)	-	-	0.456
Data from dedicated monitoring devices	18 (5.3)	13 (6.5)	2 (3.3)	-	2 (12.5)	-	1 (25.0)	0.104
Adherence reports generated by digital applications	13 (3.8)	9 (4.5)	3 (4.9)	-	1 (6.3)	-	-	0.668
**Perceived importance of the following adherence monitoring strategies (4–5 points on Likert scale)**								
Patient and/or caregiver information	318 (93.5)	188 (94.0)	56 (91.8)	52 (96.3)	14 (87.5)	4 (80.0)	4 (100)	0.578
Patient clinical record	274 (80.6)	166 (83.0)	53 (86.9)	37 (68.5)	13 (81.3)	3 (60.0)	2 (50.0)	0.050
Provider follow-up report	260 (76.5)	160 (80.0)	45 (73.8)	38 (70.4)	12 (75.0)	2 (40.0)	3 (75.0)	0.263
Local / regional information systems	202 (59.4)	120 (60.0)	40 (65.6)	29 (53.7)	9 (56.3)	1 (20.0)	3 (75.0)	0.368
Department evaluation report	183 (53.8)	117 (58.5)	34 (55.7)	21 (38.9)	8 (50.0)	1 (20.0)	2 (50.0)	0.105
Dedicated online platform	182 (53.5)	102 (51.0)	45 (73.8)	21 (38.9)	11 (68.8)	1 (20.0)	2 (50.0)	0.002
Electronic health record	165 (48.5)	96 (48.0)	35 (57.4)	20 (37.0)	11 (68.8)	1 (20.0)	2 (50.0)	0.107
Dedicated app	161 (47.4)	86 (43.0)	37 (60.7)	25 (46.3)	11 (68.8)	-	2 (50.0)	0.021
Scientific society database	114 (33.5)	66 (33.0)	27 (44.3)	11 (20.4)	8 (50.0)	-	2 (50.0)	0.031

*Other includes professionals in non-clinical roles (e.g. healthcare managers, freelance experts affiliated with scientific societies).

Notes: p-value less than 0.05 was considered statistically significant.

Table S4. Adherence interventions according to benchmarking survey participants (survey section 4).

Section on adherence interventions	Overall n (%)	Nurse n (%)	Pharmacist n (%)	Clinician n (%)	Patient representativ en (%)	Researcher /Educator n (%)	Other* n (%)	*p-value*
**Respondents**	**340**	**200 (58.8)**	**61 (17.9)**	**54 (15.9)**	**16 (4.7)**	**5 (1.5)**	**4 (1.2)**	
**Perceived importance of the following adherence interventions (4–5 points on Likert scale)**								
Involving family members or caregivers in adherence	321 (94.4)	192 (96.0)	57 (93.4)	50 (92.6)	14 (87.5)	5 (100)	3 (75.0)	0.306
Involving patients in defining therapeutic goals	307 (90.3)	183 (91.5)	51 (83.6)	51 (94.4)	14 (87.5)	4 (80.0)	4 (100.0)	0.338
Providing individual counselling	298 (87.6)	175 (87.5)	55 (90.2)	47 (87.0)	13 (81.3)	5 (100)	3 (75.0)	0.809
Tailoring the treatment plan to patients' needs	292 (85.9)	183 (91.5)	41 (67.2)	47 (87.0)	13 (81.3)	5 (100)	3 (75.0)	<0.001
Scheduling more frequent follow-up visits	256 (75.3)	160 (80.0)	40 (65.6)	37 (68.5)	14 (87.5)	3 (60.0)	2 (50.0)	0.073
Referring patients to dedicated adherence programmes	237 (69.7)	147 (73.5)	40 (65.6)	31 (57.4)	13 (81.3)	2 (40.0)	4 (100.0)	0.061
Using remote monitoring tools	210 (61.8)	123 (61.5)	42 (68.9)	28 (51.9)	12 (75.0)	2 (40.0)	3 (75.0)	0.303
Involving patients in support groups	209 (61.5)	141 (70.5)	31 (50.8)	23 (42.6)	12 (75.0)	-	2 (50.0)	<0.001
Sending SMS or email reminders and notifications	201 (59.1)	111 (55.5)	43 (70.5)	29 (53.7)	14 (87.5)	1 (20.0)	3 (75.0)	0.016
Using dedicated smartphone apps	173 (50.9)	97 (48.5)	34 (55.7)	27 (50.0)	12 (75.0)	-	3 (75.0)	0.054
**Perceived feasibility of the following adherence interventions (4–5 points on Likert scale)**								
Involving family members or caregivers in adherence	288 (84.7)	178 (89.0)	47 (77.0)	47 (87.0)	10 (62.5)	3 (60.0)	3 (75.0)	0.012
Involving patients in defining therapeutic goals	271 (79.7)	164 (82.0)	44 (72.1)	45 (83.3)	11 (68.8)	3 (60.0)	4 (100)	0.247
Tailoring the treatment plan to patients' needs	255 (75.0)	161 (80.5)	35 (57.4)	45 (83.3)	7 (43.8)	3 (60.0)	4 (100)	<0.001
Providing individual counselling	210 (61.8)	127 (63.5)	40 (65.6)	33 (61.1)	5 (31.3)	2 (40.0)	3 (75.0)	0.143
Sending SMS or email reminders and notifications	154 (45.3)	85 (42.5)	35 (57.4)	18 (33.3)	12 (75.0)	2 (40.0)	2 (50.0)	0.022
Scheduling more frequent follow-up visits	149 (43.8)	100 (50.0)	22 (36.1)	16 (29.6)	8 (50.0)	1 (20.0)	2 (50.0)	0.063
Referring patients to dedicated adherence programmes	148 (43.5)	92 (46.0)	32 (52.5)	13 (24.1)	7 (43.8)	-	4 (100.0)	0.001
Using remote monitoring tools	141 (41.5)	85 (42.5)	33 (54.1)	14 (25.9)	7 (43.8)	1 (20.0)	1 (25.0)	0.053
Involving patients in support groups	134 (39.4)	92 (46.0)	19 (31.1)	12 (22.2)	7 (43.8)	-	4 (100)	0.001
Using dedicated smartphone apps	132 (38.8)	74 (37.0)	28 (45.9)	19 (35.2)	9 (56.3)	1 (20.0)	1 (25.0)	0.418

*Other includes professionals in non-clinical roles (e.g. healthcare managers, freelance experts affiliated with scientific societies).

Notes: p-value less than 0.05 was considered statistically significant.

Table S5. Adherence barriers and unmet needs according to benchmarking survey participants (survey section 5).

Section on adherence barriers	Overall n (%)	Nurse n (%)	Pharmacist n (%)	Clinician n (%)	Patient representativen (%)	Researcher /Educator n (%)	Other n (%)	*p-value*
**Respondents**	**340**	**200 (58.8)**	**61 (17.9)**	**54 (15.9)**	**16 (4.7)**	**5 (1.5)**	**4 (1.2)**	-
**Unmet needs related to medication adherence**								
Limited patient awareness of the importance of adherence	234 (68.8)	134 (67.0)	40 (65.6)	42 (77.8)	11 (68.8)	4 (80.0)	3 (75.0)	0.701
Lack of coordination among healthcare professionals along the care pathway	205 (60.3)	115 (57.5)	44 (72.1)	31 (57.4)	9 (56.3)	4 (80.0)	2 (50.0)	0.357
Low caregiver involvement in the care process	173 (50.9)	104 (52.0)	29 (47.5)	31 (57.4)	4 (25.0)	3 (60.0)	2 (50.0)	0.331
Need for personalised and flexible adherence interventions	166 (48.8)	112 (56.0)	15 (24.6)	23 (42.6)	12 (75.0)	2 (40.0)	2 (50.0)	<0.001
Lack of specific training for healthcare professionals on adherence	162 (47.6)	98 (49.0)	30 (49.2)	21 (38.9)	7 (43.8)	4 (80.0)	2 (50.0)	0.540
Absence of structured follow-up programmes	159 (46.8)	89 (44.5)	29 (47.5)	28 (51.9)	9 (56.3)	2 (40.0)	2 (50.0)	0.892
Lack of time during patient visits	150 (44.1)	86 (43.0)	27 (44.3)	27 (50.0)	9 (56.3)	1 (20.0)	-	0.292
Limited access to monitoring technologies and remote support	145 (42.6)	85 (42.5)	30 (49.2)	23 (42.6)	5 (31.3)	1 (20.0)	1 (25.0)	0.627
Lack of financial resources for adherence-support programmes	137 (40.3)	78 (39.0)	28 (45.9)	21 (38.9)	6 (37.5)	4 (80.0)	-	0.220
Language and cultural barriers in communication	104 (30.6)	77 (38.5)	10 (16.4)	13 (24.1)	3 (18.8)	1 (20.0)	-	0.007
Need for strategies to manage medication side effects	79 (23.2)	38 (19.0)	18 (29.5)	15 (27.8)	6 (37.5)	2 (40.0)	-	0.167
**Existing barriers to optimal medication adherence**								
High number of medicines and daily doses	235 (69.1)	134 (67.0)	44 (72.1)	45 (83.3)	6 (37.5)	4 (80.0)	2 (50.0)	0.014
Lack of clear information on prescribed therapies	220 (64.7)	135 (67.5)	39 (63.9)	29 (53.7)	11 (68.8)	3 (60.0)	3 (75.0)	0.562
Lack of awareness of the importance of adherence	211 (62.1)	127 (63.5)	39 (63.9)	30 (55.6)	10 (62.5)	3 (60.0)	2 (50.0)	0.914
Lack of family or caregiver support	209 (61.5)	125 (62.5)	28 (45.9)	43 (79.6)	7 (43.8)	4 (80.0)	2 (50.0)	0.005
Patient memory problems or poor time management	200 (58.8)	120 (60.0)	32 (52.5)	36 (66.7)	7 (43.8)	4 (80.0)	1 (25.0)	0.234

Inadequate communication and coordination among HCPs	156 (45.9%)	87 (43.5%)	35 (57.4%)	19 (35.2%)	9 (56.3%)	3 (60.0%)	3 (75.0%)	0.124
Limited availability of structured follow-up programmes	132 (38.8)	79 (39.5)	24 (39.3)	16 (29.6)	8 (50.0)	3 (60.0)	2 (50.0)	0.555
Perceived adverse effects	125 (36.8)	59 (29.5)	25 (41.0)	31 (57.4)	6 (37.5)	3 (60.0)	1 (25.0)	0.006
Language and cultural barriers to understanding instructions	121 (35.6)	89 (44.5)	11 (18.0)	17 (31.5)	2 (12.5)	1 (20.0)	1 (25.0)	0.001
Economic difficulties and limited access to medicines	109 (32.1)	75 (37.5)	8 (13.1)	17 (31.5)	7 (43.8)	2 (40.0)	-	0.007
Lack of trust in medicines and healthcare	103 (30.3)	59 (29.5)	13 (21.3)	25 (46.3)	5 (31.3)	1 (20.0)	-	0.053
Limited access to counselling or psychological support services	67 (19.7)	46 (23.0)	5 (8.2)	6 (11.1)	8 (50.0)	1 (20.0)	1 (25.0)	0.003

*Other includes professionals in non-clinical roles (e.g. healthcare managers, freelance experts affiliated with scientific societies).

Notes: p-value less than 0.05 was considered statistically significant.

Table S6. Unmet needs and barriers endorsed by benchmarking respondents, stratified by Italian macro-area (North, Centre, South).

Domain and Item	P value test	Cramér's V test	Northern Italy	Central Italy	Southern Italy
**Barriers to adherence**					
Other	0.801	0.029	2/210 (1.0%)	1/58 (1.7%)	1/70 (1.4%)
Lack of family support or carers	0.001	0.205	146/210 (69.5%)	30/58 (51.7%)	33/70 (47.1%)
Language and cultural barriers to understanding instructions	<0.001	0.232	92/210 (43.8%)	16/58 (27.6%)	12/70 (17.1%)
Financial difficulties and limited access to medicines	0.008	0.168	62/210 (29.5%)	14/58 (24.1%)	33/70 (47.1%)
Limited availability of structured follow-up programmes	0.934	0.020	83/210 (39.5%)	23/58 (39.7%)	26/70 (37.1%)
High number of medicines and daily doses	0.001	0.203	158/210 (75.2%)	40/58 (69.0%)	36/70 (51.4%)
Inadequate communication and coordination between healthcare professionals	0.034	0.141	87/210 (41.4%)	35/58 (60.3%)	34/70 (48.6%)
Limited access to counselling or psychological support services	0.019	0.153	40/210 (19.0%)	6/58 (10.3%)	21/70 (30.0%)
Lack of awareness regarding the importance of adherence	0.217	0.095	138/210 (65.7%)	35/58 (60.3%)	38/70 (54.3%)
Lack of clear information on prescribed treatments	0.013	0.160	145/210 (69.0%)	28/58 (48.3%)	46/70 (65.7%)
Perception of side effects	0.050	0.133	88/210 (41.9%)	18/58 (31.0%)	19/70 (27.1%)
Memory or time management issues on the part of the patient	0.141	0.108	130/210 (61.9%)	35/58 (60.3%)	34/70 (48.6%)
Distrust of medicine and medication	0.967	0.014	64/210 (30.5%)	17/58 (29.3%)	22/70 (31.4%)
**Unmet needs**					
Limited access to remote monitoring and support technologies	0.788	0.038	93/210 (44.3%)	23/58 (39.7%)	29/70 (41.4%)
Other	1.000	0.047	2/210 (1.0%)	0/58 (0.0%)	1/70 (1.4%)
Lack of structured follow-up programmes	0.811	0.035	96/210 (45.7%)	29/58 (50.0%)	34/70 (48.6%)
Language and cultural barriers in communication	0.041	0.137	75/210 (35.7%)	13/58 (22.4%)	16/70 (22.9%)
Need for strategies to manage the side effects of medicines	0.649	0.051	52/210 (24.8%)	11/58 (19.0%)	16/70 (22.9%)
Limited patient awareness of the importance of adherence	0.176	0.101	151/210 (71.9%)	40/58 (69.0%)	42/70 (60.0%)
Lack of coordination between different healthcare professionals throughout the care pathway	0.236	0.092	120/210 (57.1%)	39/58 (67.2%)	46/70 (65.7%)
Lack of specific training for healthcare professionals on adherence	0.337	0.080	94/210 (44.8%)	32/58 (55.2%)	35/70 (50.0%)

Lack of financial resources for support programmes	0.022	0.151	73/210 (34.8%)	29/58 (50.0%)	35/70 (50.0%)
Lack of time during patient consultations	0.043	0.136	102/210 (48.6%)	25/58 (43.1%)	22/70 (31.4%)
Need for personalised and flexible interventions	0.136	0.109	112/210 (53.3%)	25/58 (43.1%)	29/70 (41.4%)
Low involvement of carers in the care process	0.430	0.071	111/210 (52.9%)	31/58 (53.4%)	31/70 (44.3%)

Footnote: Values are n/N (%), where N is the number of respondents with non-missing data for the item. P-values are from a χ^2 test of independence; Fisher's exact test was used when expected cell counts were <5. Two-sided $p<0.05$ was considered statistically significant. Cramér's V quantifies the strength of association between Italian macro-area and item endorsement (0 = no association; 1 = perfect association).

Table S7. Unmet needs and barriers endorsed by benchmarking respondents, stratified by care setting.

Domain and Items	P value test	Cramér's V test	Academic	Hospital	Other	Pharmacy	Primary / Territorial care
**Barriers to adherence**							
Other	0.275	0.104	0/7 (0.0%)	2/192 (1.0%)	1/19 (5.3%)	0/61 (0.0%)	1/61 (1.6%)
Lack of family support or carers	0.034	0.175	5/7 (71.4%)	126/192 (65.6%)	9/19 (47.4%)	28/61 (45.9%)	41/61 (67.2%)
Language and cultural barriers to understanding instructions	<0.001	0.237	2/7 (28.6%)	77/192 (40.1%)	2/19 (10.5%)	11/61 (18.0%)	29/61 (47.5%)
Financial difficulties and limited access to medicines	0.005	0.194	3/7 (42.9%)	68/192 (35.4%)	8/19 (42.1%)	8/61 (13.1%)	22/61 (36.1%)
Limited availability of structured follow-up programmes	0.774	0.072	4/7 (57.1%)	72/192 (37.5%)	9/19 (47.4%)	24/61 (39.3%)	23/61 (37.7%)
High number of medicines and daily doses	0.151	0.144	5/7 (71.4%)	136/192 (70.8%)	8/19 (42.1%)	44/61 (72.1%)	42/61 (68.9%)
Inadequate communication and coordination between healthcare professionals	0.015	0.188	5/7 (71.4%)	73/192 (38.0%)	11/19 (57.9%)	35/61 (57.4%)	32/61 (52.5%)
Limited access to counselling or psychological support services	0.002	0.221	2/7 (28.6%)	35/192 (18.2%)	9/19 (47.4%)	5/61 (8.2%)	16/61 (26.2%)
Lack of awareness regarding the importance of adherence	0.945	0.046	4/7 (57.1%)	116/192 (60.4%)	12/19 (63.2%)	39/61 (63.9%)	40/61 (65.6%)
Lack of clear information on prescribed treatments	0.951	0.048	5/7 (71.4%)	126/192 (65.6%)	13/19 (68.4%)	39/61 (63.9%)	37/61 (60.7%)
Perception of side effects	0.729	0.077	4/7 (57.1%)	68/192 (35.4%)	7/19 (36.8%)	25/61 (41.0%)	21/61 (34.4%)
Memory or time management issues on the part of the patient	0.198	0.134	5/7 (71.4%)	119/192 (62.0%)	7/19 (36.8%)	32/61 (52.5%)	37/61 (60.7%)
Distrust of medicine and medication	0.388	0.111	2/7 (28.6%)	66/192 (34.4%)	5/19 (26.3%)	13/61 (21.3%)	17/61 (27.9%)
**Unmet needs**							
Limited access to remote monitoring and support technologies	0.451	0.106	2/7 (28.6%)	83/192 (43.2%)	5/19 (26.3%)	30/61 (49.2%)	25/61 (41.0%)
Other	0.821	0.060	0/7 (0.0%)	2/192 (1.0%)	0/19 (0.0%)	0/61 (0.0%)	1/61 (1.6%)
Lack of structured follow-up programmes	0.757	0.074	3/7 (42.9%)	93/192 (48.4%)	10/19 (52.6%)	29/61 (47.5%)	24/61 (39.3%)
Language and cultural barriers in communication	0.011	0.191	2/7 (28.6%)	63/192 (32.8%)	3/19 (15.8%)	10/61 (16.4%)	26/61 (42.6%)
Need for strategies to manage the side effects of medicines	0.278	0.117	3/7 (42.9%)	40/192 (20.8%)	6/19 (31.6%)	18/61 (29.5%)	12/61 (19.7%)

Limited patient awareness of the importance of adherence	0.904	0.062	6/7 (85.7%)	132/192 (68.8%)	13/19 (68.4%)	40/61 (65.6%)	43/61 (70.5%)
Lack of coordination between different healthcare professionals throughout the care pathway	0.269	0.123	5/7 (71.4%)	111/192 (57.8%)	10/19 (52.6%)	44/61 (72.1%)	35/61 (57.4%)
Lack of specific training for healthcare professionals on adherence	0.251	0.127	6/7 (85.7%)	93/192 (48.4%)	8/19 (42.1%)	30/61 (49.2%)	25/61 (41.0%)
Lack of financial resources for support programmes	0.242	0.129	5/7 (71.4%)	78/192 (40.6%)	6/19 (31.6%)	28/61 (45.9%)	20/61 (32.8%)
Lack of time during patient consultations	0.910	0.057	2/7 (28.6%)	87/192 (45.3%)	9/19 (47.4%)	27/61 (44.3%)	25/61 (41.0%)
Need for personalised and flexible interventions	<0.001	0.245	3/7 (42.9%)	99/192 (51.6%)	13/19 (68.4%)	15/61 (24.6%)	36/61 (59.0%)
Low involvement of carers in the care process	0.687	0.082	4/7 (57.1%)	102/192 (53.1%)	7/19 (36.8%)	29/61 (47.5%)	31/61 (50.8%)

Footnote: Values are n/N (%), where N is the number of respondents with non-missing data for the item. P-values are from a χ^2 test of independence; Fisher's exact test was used when expected cell counts were <5. Two-sided $p<0.05$ was considered statistically significant. Cramér's V quantifies the strength of association between professional setting and item endorsement (0 = no association; 1 = perfect association).

Table S8. Agreement rates and consensus levels for SWOT items (proportion of respondents scoring 4–5 on the 5-point Likert scale).

Item	Respondents with Likert score 4–5, n/N*	Agreement (%; scores 4–5)	Consensus status (≥50% agreement)
**Strengths**			
The direct involvement of healthcare professionals (physicians, nurses, pharmacists) in consultations with patients effectively supports medication adherence in my care setting.	9/14	64.3%	**Moderate**
In my care setting, good practices for the management of medication adherence have already been implemented.	6/14	42.9%	No
Effective technological tools are available for monitoring medication adherence.	4/14	28.6%	No
There is strong collaboration between different healthcare professionals.	2/14	14.3%	No
The availability of clinical and prescribing data is sufficient to enable effective monitoring of medication adherence.	2/14	14.3%	No
Human resources for the management of medication adherence (qualified personnel) are adequate.	0/13	-	No
**Weaknesses**			
There are no incentives for healthcare professionals to improve the management of medication adherence.	10/14	71.4%	**Moderate**
Financial resources allocated to medication adherence support programmes, such as educational campaigns, patient monitoring, and training of healthcare professionals, are insufficient in my care setting.	9/14	64.3%	**Moderate**
The use of wearable devices and digital applications to monitor medication adherence is still insufficient in my care setting.	9/14	64.3%	**Moderate**
There are gaps in the training of healthcare professionals on medication adherence.	8/14	57.1%	**Moderate**
Poor coordination among different healthcare professionals along the care pathway represents a significant barrier to the management of medication adherence.	8/14	57.1%	**Moderate**
Patients' access to some medicines is limited by factors such as availability, distribution, or associated costs.	7/13	53.8%	**Moderate**
Patients are not sufficiently involved in defining therapeutic goals, which limits their medication adherence.	6/14	42.9%	No
Patients do not receive adequate or understandable information about prescribed therapies, which negatively affects medication adherence.	4/14	28.6%	No
**Opportunities**			
Increasing patients' awareness of the importance of medication adherence through educational programmes may lead to significant improvements.	12/14	85.7%	**Strong**
Establishing collaborations with patient organisations/associations to enhance awareness about medication adherence.	12/14	85.7%	**Strong**
Involving family members or caregivers in defining and monitoring therapeutic goals.	12/14	85.7%	**Strong**
Promoting the use of digital technologies for monitoring medication adherence.	12/14	85.7%	**Strong**
Facilitating access to dedicated funding to develop and implement interventions aimed at improving medication	11/14	78.6%	**Strong**

adherence (e.g. monitoring programmes, patient support, information for healthcare professionals).			
Implementing improvement strategies within regional health policies to support medication adherence.	11/14	78.6%	**Strong**
Strengthening the role of nurses and general practitioners in monitoring medication adherence could substantially improve its management.	11/14	78.6%	**Strong**
Implementing new training programmes for healthcare professionals.	10/14	71.4%	**Moderate**
**Threats**			
Frequent changes in regional health policies hinder the continuity and effectiveness of interventions aimed at improving medication adherence.	10/13	76.9%	**Moderate**
Patients have limited awareness of the importance of correctly following their therapies, which hinders the success of adherence interventions.	10/14	71.4%	**Moderate**
Financial resources allocated to medication adherence programmes are scarce, limiting the possibility of implementing effective and sustainable interventions.	10/14	71.4%	**Moderate**
The high number of medicines and the complexity of therapeutic regimens represent a barrier to medication adherence for patients.	9/14	64.3%	**Moderate**
The limited availability of psychological support and counselling services represents a barrier for patients in maintaining medication adherence.	8/14	57.1%	**Moderate**
There is a lack of support from local institutions.	6/14	42.9%	No

* N varies due to missing.

Notes: Items are 5-point Likert-scale closed questions derived from the results of the benchmarking survey. Items that reached consensus in this table were used to generate deductive codes. Consensus was defined a priori as ≥50% of respondents selecting 4 (“agree”) or 5 (“strongly agree”) on the 5-point Likert scale. Agreement between 50–74% was classified as “moderate consensus”, and ≥75% as “strong consensus”. Inductive codes were then developed from participants’ open-ended responses.

Table S9. Summary of thematic matrices and generative codes from the SWOT analysis.

Thematic matrix	Code type ID	Code label	Respondent n/N* (%)
**Strengths**			
Communication and relationship with patient	IC1	Direct patient–provider communication	1/14 (7.1)
	IC6	Continuity of the therapeutic relationship	1/14 (7.1)
Multiprofessional roles and coordination	DC1	Direct involvement of healthcare professionals	9/14 (64.3)
	IC4	Nurse-led coordination of adherence-related activities	2/14 (14.3)
	IC5	Collaboration with clinical pharmacology and trainees	2/14 (14.3)
Education and monitoring	IC2	Therapeutic education	1/14 (7.1)
	IC3	Clinical and therapeutic monitoring	2/14 (14.3)
**Weaknesses**			
Insufficient communication and relationship with patients	IC1	Limited patient–provider communication	1/14 (7.1)
Lack of interprofessional collaboration and communication	DC1	Poor coordination among healthcare professionals along the care pathway	8/14 (57.1)
	DC3	Gaps in training of healthcare professionals	8/14 (57.1)
	IC6	Weak networking and integration of available services	1/14 (7.1)
Insufficient financial and material resources	DC4	Limited access to some medicines (availability, distribution, or costs)	7/13 (53.8)
	DC5	Insufficient financial resources invested in adherence-support programmes	9/14 (64.3)
	DC6	Lack of incentives for healthcare professionals to improve adherence management	10/14 (71.4)
	IC2	Scarcity of resources and tools for patient monitoring	1/14 (7.1)
Inadequate time for appropriate adherence management	IC4	Workload pressure and limited time for adherence-related activities	2/14 (14.3)
	IC5	Short hospital stays limiting adherence counselling	1/14 (7.1)
Limited use of technologies and digitalisation	DC2	Limited use of digital tools and wearables for adherence monitoring	9/14 (64.3)
**Opportunities**			
Education and training for healthcare professionals and patients	DC1	Continuous training for healthcare professionals	10/14 (71.4)
	DC2	Patient health education	12/14 (85.7)
	IC1	Integration of education and training initiatives	3/14 (21.4)
	IC2	Training opportunities for healthcare professionals	2/14 (14.3)
Patient engagement and empowerment	DC5	Involvement of family members or caregivers	12/14 (85.7)
	DC3	Collaboration with patient associations	12/14 (85.7)
	IC4	Broader engagement of patients and caregivers	1/14 (7.1)
Strengthening healthcare professionals' role in adherence management	DC4	Enhanced role of general practitioners and nurses in adherence management	11/14 (78.6)
	IC3	Interprofessional collaboration and enhanced nursing role	1/14 (7.1)
Policy-driven promotion of resources and digital technologies	DC6	Promotion of digital technologies for adherence monitoring	12/14 (85.7)
	DC7	Access to dedicated funding for adherence-improvement interventions	11/14 (78.6)
	DC8	Improvement of regional health policies to support adherence	11/14 (78.6)

Threats			
Therapeutic complexity and prescribing inappropriateness	DC1	Therapeutic regimen complexity and polypharmacy	9/14 (64.3)
	IC2	Therapeutic complexity and inappropriate prescribing	2/14 (14.3)
Limited specific and relational training	IC1	Inadequate relational preparation of healthcare professionals	1/14 (7.1)
Limited patient support	DC2	Low patient awareness of the importance of adherence	10/14 (71.4)
	DC3	Limited availability of psychological support and counselling services	8/14 (57.1)
Limited multidisciplinary collaboration	IC3	Poor multidisciplinary collaboration in clinical practice	3/14 (21.4)
Limited political-institutional support	DC4	Insufficient financial resources for adherence programmes	10/14 (71.4)
	DC5	Lack of continuity of local health policies	10/13 (76.9)
	IC4	Lack of political-institutional support for adherence initiatives	1/14 (7.1)

* N varies due to missing.

Abbreviations: DC, deductive code; IC, inductive code.

Notes: Frequencies indicate the number and proportion of participants who contributed to each code. For deductive codes, frequencies correspond to the number of respondents who agreed with the originating closed-ended item (Likert score 4–5). For inductive codes, frequencies represent the number of participants whose open-ended responses were coded under that theme.
